# Comparison of clinical characteristics of patients with adductor laryngeal dystonia in the focal and segmental types

**DOI:** 10.1590/S1808-86942011000400002

**Published:** 2015-10-19

**Authors:** Gustavo Polacow Korn, Miriam Moraes, Luiz Celso Pereira Vilanova, Bruno Teixeira de Moraes, Glaucya Madazio, Marina Padovani, Noemi Grigoletto De Biase

**Affiliations:** 1 Medical doctor, doctoral degree in sciences, Otorhinolaryngology and Head & Neck Surgery Department, São Paulo Federal University, UNIFESP; 2 Speech therapist, master's degree, specialist in human communications disorders, São Paulo Federal University, UNIFESP. Expert on voice, CEV; 3 Medical doctor, Neurology Department, São Paulo Federal University, UNIFESP. Associate professor in the Neurology Department, São Paulo Federal University, UNIFESP; 4 Medical doctor, graduate student in the Otorhinolaryngology and Head & Neck Surgery Department, São Paulo Federal University, UNIFESP; 5 Speech therapist, expert on voice. Doctoral degree, Human Communications Science Department. Speech therapist, São Paulo Federal University, UNIFESP. Professor of the Specialization Course on Voice, CEV; 6 Speech therapist, expert on voice. Master's degree. Human Communications Science Department. Speech therapist, São Paulo Federal University, UNIFESP. Graduate student, Human Communications Science Department. Speech therapist, São Paulo Federal University, UNIFESP; 7 Medical doctor, associate professor in Otorhinolaryngology and Head & Neck Surgery Department, São Paulo Federal University, UNIFESP. Collaborator in the Otorhinolaryngology and Head & Neck Surgery Department, São Paulo Federal University, UNIFESP. Associate professor, speech therapy course, PUC-SP

**Keywords:** dysphonia, dystonic disorders, meige syndrome

## Abstract

**Abstract:**

Dystonia is a central motor processing neurological disorder characterized by abnormal, often action-induced, involuntary movements or uncontrolled spasms.

**Aim:**

To compare patients with the diagnoses of focal and segmental adductor laryngeal dystonia at the Neurolarynx Outpatient Clinic of the Federal University of São Paulo.

**Materials and methods:**

A clinical retrospective study of data collected from patient registries from 2003 to 2009.

**Results:**

Of 34 patients, 25 presented focal dystonia and 9 presented segmental dystonia. There were 30 females (88.2%) and 4 males (11.8%). A relation with a traumatic event was reported in 11 cases (32.4%). Vocal tremor was observed in 21 patients (61.8%). The mean age at onset, the age at diagnosis, and time between the onset and the diagnosis were respectively 55, 61.3 and 6.3 years. There was no statistical difference between patients with focal laryngeal adductor dystonia and segmental dystonia in the study data.

**Conclusion:**

There were no statistical differences among patients with focal adductor laryngeal dystonia and segmental dystonia relating to age of onset, age of diagnosis, gender, time between onset and diagnosis, presence of associated tremor, and relation to trauma.

## INTRODUCTION

Dystonia is a central motor processing neurological disorder^1^. It consists of involuntary movements resulting from sustained muscle contractions that result in torsion, repetitive movements, or abnormal postures, which may affect any part of the body^2^.

Depending on the affected muscles, dystonia may be focal (involving a muscle group), segmental (a muscle segment), multifocal (non-adjacent muscle groups), hemidystonic, or generalized. The etiology may be primary or secondary. In primary dystonia, the clinical examination and neuroimaging reveal no brain lesions; in this case, it may be sporadic or hereditary^1^. Secondary dystonia results from brain injury of several causes.

Data from Columbia University have shown that the onset of dystonia may occur at any age, ranging from 9 months to 85 years. The peak onset of generalized dystonia occurs around 10 years of age; it is around ages 45 to 60 in segmental dystonia, and around 45 years of age in focal dystonia. The peak onset of focal laryngeal dystonia ranges from 35 to 50 years. Hereditary or idiopathic generalized dystonia almost always presents initially as focal dystonia before affecting other bodily segments^3^.

Cranial segmental dystonia may affect the eyelid muscles (blepharospasm), the oromandibular muscles (affecting the perioral and chewing muscles, and the tongue), and the larynx. Thus, speech may be affected because of extralaryngeal dystonia involving the joints, or secondary to dystonia affecting the voice tract and altering resonance^4^.

Laryngeal dystonia or spasmodic dysphonia are the names given when dystonia affects the larynx; it is characterized by abnormal involuntary movements of the vocal folds, and is therefore one of the most incapacitating voice disorders^5,^^6^.

Laryngeal dystonia may be classified as: adduction laryngeal dystonia, abduction laryngeal dystonia, mixed laryngeal dystonia, and respiratory laryngeal dystonia^5,^^7^. The first three affect phonation. The respiratory form is rare, but raises concern because there are paradoxical movements of vocal folds during inspiration^8,^^9^, which may restrict the passage of air and become a medical emergency.

Adduction laryngeal dystonia is the most frequent form; the adductor muscles contract significantly during phonation, resulting in inappropriate hyperadcuction^8^. The voice takes on a characteristic strain-strangled quality, with frequent breaks in sonority and evident effort^3,^^10^. Tremor may accompany all forms^9,^^11^.

The diagnosis of laryngeal dystonia is made clinically: a perceptive-auditory voice assessment and nasofibrolaryngoscopy^8,^^10^. Diagnostic tests, such as weak and strong utterance of high and low pitch sounds, and phrases where muted or sonorous phonemes predominate, help in the differential diagnosis of laryngeal dystonia^10^. Electromyography may help confirm the diagnosis, especially the respiratory forms^8,^^9,^^12^.

Knowledge of the clinical features of patients with laryngeal dystonia may facilitate the diagnosis, treatment, planning of care, health-promoting measures, and improve the quality of life of these patients.

## OBJECTIVE

The purpose of this study was to compare patients with focal and segmental adduction laryngeal dystonia, seen at the Voice and Larynx Sector - Neurolaryngeal Outpatient Unit of the Voice and Larynx Interdisciplinary Clinic of a tertiary hospital, Sao Paulo Federal University.

## METHOD

A retrospective study was made of the registries of 34 male and female patients seen at the Voice and Larynx Sector - Neurolaryngeal Outpatient Unit of the Voice and Larynx Interdisciplinary Clinic of a tertiary hospital, Sao Paulo Federal University (UNIFESP) from 2003 to 2009. Patients were diagnosed with laryngeal dystonia; 25 had focal adduction dystonia and 9 had segmental dystonia. All patients sought the clinic voluntarily for an evaluation and treatment of dysphonia, and all underwent otorhinolaryngological, phonoaudiological, and neurological assessments.

Comparative data among groups were: age of onset of complaints, age at diagnosis, duration of symptoms until the diagnosis was made, presence of associated tremor, and relation with traumatic situations.

Adduction dystonia was classified according to the symptoms, perceptive-auditory voice assessment^13^, fibronasolaryngoscopy^8^, and electromyography.

The otorhinolaryngological evaluation consisted of fibronasolaryngoscopy for observing the vocal folds at rest, respiration, phonation, a functional study of voice production and non-phonatory tasks. Phonation testing included sustained uttering of the vowels /e/ in usual tones, the vowel /i/ at high pitches, the vowel /e/ in ascending and descending glissando, strong and weak intensity utterances, whispered utterances, and phrases where muted and sonorous sounds predominated. Adduction dystonia manifests spasm when the larynx participates in utterances and when the vocal folds meet, that is, mostly when sonorous sounds are uttered; spasm decrease or disappear when treble and muted sounds are uttered.

A neurologist also evaluated all patients after the otorhinolaryngologic assessment.

A speech therapist evaluated voice behavior and the perceptive-auditory voice assessment. The speech material consisted of sustained utterance of the vowel “é”. In this task, voice quality changes become more evident than in connected speech, when subjects may mask voice quality and the deviation degree of voice changes. The evaluation was a consensus among thee speech therapists that specialize in voice; voice assessment took place when patients were admitted to the Neurolaryngeal Outpatient Unit.

Patients were offered botulinum toxin therapy on the left vocal fold (eight units, with electromyography).

Data were summarized as the mean, standard deviation, minimum, median, and maximum for numeric variables; categorical variables were expressed as frequencies and percentages. The following variables were compared among the focal and segmental dystonia groups: age at the onset of complaints, sex, age at the diagnosis, time elapsed from the beginning of symptoms to the diagnosis, associated tremor, relation with traumatic situations.

Fisher's exact test was applied for comparing the variables sex, associated tremor, and relation with traumatic situations, in the focal and segmental dystonia groups.

The ANOVA model was applied for comparing the variables age at the onset of complaints, age at diagnosis, and duration of complaints until the diagnosis, in the focal and segmental dystonia groups. The logarithmic transformation was applied to the variable duration of compliant until the diagnosis to ensure the normalcy of this variable. The significance level was 5%.

The institutional review board of the Sao Paulo Federal University approved this study (protocol 0029/05).

## RESULTS

There were 34 patients with laryngeal dystonia, of which 25 had focal laryngeal dystonia and 9 had segmental dystonia.

The mean age of onset of complaints was 55 years; the mean age at the diagnosis was 61.3 years, and the time elapsed from the beginning of symptoms to the diagnosis was 6.3 years.

The focal and segmental dystonia groups did not differ significantly in any of the variables at a 5% significance level (Table 1).

**Table 1:** Descriptive analysis per group (focal X segmental dystonia) for the variables: current age, age at diagnosis, and time elapsed from the first complaint to the diagnosis.

Variables per group	Mean	Standard deviation	Minimum	Median	Maximum	Total	*p*-value
**Age at onset of complaints**							
Focal laryngeal	56.5	13.2	30.0	60.0	75.0	25	
Segmental	50.6	12.2	24.0	52.0	66.0	9	0.244
**Age at the diagnosis**							
Focal laryngeal	61.8	12.2	34.0	66.0	76.0	25	
Segmental	59.7	14.4	25.0	63.0	72.0	9	0.665
**Time elapsed from the complaint to the diagnosis (in years)**							
Focal laryngeal	5.3	5.3	0.5	3.0	20.0	25	
Segmental	9.1	9.0	1.0	4.0	23.0	9	0.253

*p*-value, ANOVA model.

There were 30 females (88.2%) and 4 males (11.8%) in the study sample. A relation with traumatic situations was found in 11 cases (32.4%).

Table 2 presents the data showing that there were no statistically significant differences in gender or relation with traumatic situations between the focal and segmental dystonia groups.

**Table 2:** Descriptive analysis per group (focal X segmental dystonia) for the variables: sex and relation with traumatic situations

Variables	Focal dystonia Total (%)	Segmental Total (%)	*p*-value
**Sex**			
Male	2 (8.0%)	2 (22.2%)	
Female	23 (92.0%)	7 (77.8%)	0.281
**Relation with traumatic situation**			
No	19 (76.0%)	4 (44.4%)	
Yes	6 (24.0%)	5 (55.6%)	0.111

*p*-value - Fisher's exact test.

Tremor associated with dystonia was found in 21 patients (61.8%).

The results presented on Table 3 show that the focal and segmental dystonia groups did not differ in relation to the variable tremor, at a 5% significance level.

**Table 3:** Descriptive analysis per group (focal X segmental dystonia) for associated tremor.

Associated tremor	Focal dystonia Total (%)	Segmental Total (%)	*p*-value
No	9 (36.0%)	4 (44.4%)	
Yes	16 (64.0%)	5 (55.6%)	0.704

*p*-value - Fisher's exact test.

## DISCUSSION

A comparison between focal and segmental laryngeal dystonia revealed no statistically significant differences in the age of onset of symptoms, the age at the diagnosis, the time elapsed from the beginning of symptoms to the diagnosis, sex, relation with traumatic situations, and presence of tumors (Tables 1, 2 and 3).

The mean age of onset of symptoms in the focal and segmental laryngeal groups was in turn 56 and 50 years; the difference was not statistically significant (Table 1). Brin et al^3^. noted that the onset of focal laryngeal dystonia peaked between 35 and 50 years, and that of segmental laryngeal dystonia peaked between 45 and 60 years; these authors, however did not make a statistical analysis of the difference between both groups. We believe that our small study sample explains the absence of onset age differences in the focal and segmental laryngeal dystonia groups. There are no published studies comparing other aspects between these two groups.

The mean age at which symptoms began - around 55 years (Table 1) - is higher than the mean age reported by Blitzer et al^8^. (39 years). The mean age at the diagnosis in focal cases was 61.8 years, close to the 56 year value noted by Tisch et al^14^.

The diagnosis of focal and segmental laryngeal dystonia was generally made after 5 and 9 years respectively (median - 3 and 4 years respectively) from the onset of symptoms (Table 1); there were cases in which the diagnosis was made 23 years after the onset of symptoms. As most patients usually first visit a general practitioner, this delay may be partly explained by lack of knowledge about this uncommon condition, which still has many unclear features. It should be noted that the above mentioned author is a reference expert at a major center that receives patients from several distant localities, and therefore has a significant series (901 patients with laryngeal dystonia by 1998)^8^. Many patients seen at the Neurolaryngeal Outpatient Unit of the tertiary XXX Hospital are referred from other medical units and specialties; thus, deeper knowledge about dystonia could result in earlier diagnoses and treatment, thereby improving the quality of life of patients. The time elapsed between the complaint and the diagnosis ranged from 6 months to 20 years (mean - 6 years) (Table 1), a relatively long period compared to the 2 months reported by Aronson^15^, and especially when considering an essential function for social life and communication. These patients may become withdrawn at especially important periods of their lives. Laryngeal dystonia may affect the emotional, social, and occupational aspects of patient's lives, depending on the circumstances or any premorbid personality disorder^15^.

Blitzer et al.^8^ found focal dystonia (66.1%) and segmental dystonia (28.5%) in primary cases; our findings are similar to these values (focal - 73.5%; segmental -26.5%). These authors also added that other bodily segments in 16% of focal laryngeal dystonia patients may be involved. Koufman & Blalock^5^ found a 3% rate of non-focal cases (these were not specified in the article as segmental or generalized).

Females predominated in our sample (88.2%, Table 2). Blitzer et al.^8^ also noted a female predominance (63% of 744 primary cases), as did Klotz et al.^16^ (74.3% of 214 patients), Elmiyeh et al.^17^ (62% of 68 patients), and Ludlow et al.^18^ (93.7% of 16 patients). The same applied in other studies of laryngeal dystonia in which adduction laryngeal dystonia predominated, such as in Tisch et al.^14^ (62.1% of 169 patients), Schweinfurth et al.^19^ (79% of 168 patients), Soland et al.^20^ (72.2% of 36 cases), and Adler et al.^21^ (79.3% of 270 patients).

Associated tumors were present in 64% of focal cases and 55.6% of segmental cases (Table 3); these values were similar to those of Koufman & Blalock^5^ (59%), but higher than those of Blitzer et al.^22^ (29%).

A relation with traumatic situations was 32.4% in our study sample, ranging from 24% (focal) to 55.6% (segmental) (Table 2); the difference was not statistically significant. This value was close to the percentage Brodnitz^23^ observed (40%). This explains, at least partly, our initial psychogenic hypothesis. Stress originating from a primary psychogenic cause and stress as a predisposing factor or a trigger for a latent neurological disorder need to be distinguished^15^. The fact that positive or negative emotions, stress, and psychological factors have an effect on the larynx and laryngeal diseases is well known. Further studies are needed to clarify the pathogenesis of dystonia and the eventual role of traumatic situations on this dysfunction.

Our findings in this study about adduction laryngeal dystonia patients are similar to those in previously published studies except for the time elapsed from the first symptoms and the diagnosis^15^, and the percentage of segmental cases, in comparison with one of the authors^5^.

Our data show that there are no statistically significant differences in all of the aspects that were investigated in both focal and segmental dystonia.

## CONCLUSION

There were no differences between patients with focal laryngeal dystonia and segmental laryngeal dystonia with respect to age of onset, age at diagnosis, gender, time elapsed between the first symptoms and the diagnosis, the presence of associated tumors, and relation with traumatic situations.
